# Targeted Therapies in Cancer: To Be or Not to Be, Selective

**DOI:** 10.3390/biomedicines9111591

**Published:** 2021-11-01

**Authors:** Skye Montoya, Deborah Soong, Nina Nguyen, Maurizio Affer, Sailasya P. Munamarty, Justin Taylor

**Affiliations:** Sylvester Comprehensive Cancer Center, Miller School of Medicine, University of Miami, 1501 NW 10th Avenue, Miami, FL 33136, USA; osg16@miami.edu (S.M.); deborah.soong@jhsmiami.org (D.S.); nina.nguyen@jhsmiami.org (N.N.); mxa2131@med.miami.edu (M.A.); sailasyam.13@gmail.com (S.P.M.)

**Keywords:** cancer, targeted therapies, resistance mechanisms, receptor tyrosine kinases, single kinase inhibitors, multikinase inhibitors, clinical trial inhibitors, combination drug therapies

## Abstract

Development of targeted therapies in recent years revealed several nonchemotherapeutic options for patients. Chief among targeted therapies is small molecule kinase inhibitors targeting key oncogenic signaling proteins. Through competitive and noncompetitive inhibition of these kinases, and therefore the pathways they activate, cancers can be slowed or completely eradicated, leading to partial or complete remissions for many cancer types. Unfortunately, for many patients, resistance to targeted therapies, such as kinase inhibitors, ultimately develops and can necessitate multiple lines of treatment. Drug resistance can either be de novo or acquired after months or years of drug exposure. Since resistance can be due to several unique mechanisms, there is no one-size-fits-all solution to this problem. However, combinations that target complimentary pathways or potential escape mechanisms appear to be more effective than sequential therapy. Combinations of single kinase inhibitors or alternately multikinase inhibitor drugs could be used to achieve this goal. Understanding how to efficiently target cancer cells and overcome resistance to prior lines of therapy became imperative to the success of cancer treatment. Due to the complexity of cancer, effective treatment options in the future will likely require mixing and matching these approaches in different cancer types and different disease stages.

## 1. Introduction

The field of targeted therapies in cancer is still relatively young and many questions remain unanswered. After initially moving away from chemotherapy to focus on more selective targeted therapies, the proverbial pendulum possibly swung too far as resistance to these agents is being recognized and grappled with. Kinase inhibitors as a class are among the most developed therapies offered today and serve as the prototypical small molecule targeted therapy. Protein kinases are enzymes that catalyze the transfer of the γ-phosphate group from an ATP molecule to protein residues containing a hydroxyl group, initiating a cascade of events affecting multiple downstream targets [1]. Through competitive and noncompetitive inhibition of these kinases, and therefore the pathways they activate, cancers can be slowed or completely eradicated, leading to partial or complete remissions for many cancer types [2]. However, as a monotherapy, many kinase inhibitors require continuous therapy to keep cancer in remission [1,2,3]. Despite numerous advancements made in the development of targeted therapies, many patients still face relapse due to drug resistance, which can be either de novo, a genetic alteration or variant causing resistance prior to being treated, or acquired, e.g., a resistant variant that develops after being introduced to a drug [3].

Patients with advanced or metastatic cancer may end up receiving multiple lines of treatment over the course of their disease [1,2,3,4]. Understanding how to efficiently target cancer cells and overcome resistance to prior lines of therapy became imperative to the success of cancer treatment. Kinase targeting cancer therapies can be selective (only targeting one kinase in a pathway), nonselective (targeting multiple kinases simultaneously), or can be used in combination with other cancer therapeutics: two selective kinase inhibitors working synergistically to allow a stronger effect against the tumor with less toxicity for the patient [5]. While specificity sounds attractive for drug development, most cancers have multiple aberrant pathway activation and can often find simple ways to evade very selective targeted inhibition [2,3,4,5,6]. In this review, we will discuss the advantages and disadvantages of different potential treatment strategies available today as well as some future options that are beginning to enter clinical trials. This review is not meant to be an exhaustive list of all targeted therapies available but a concise and comprehensive set of examples of different treatment strategies to discuss the pros and cons of each.

## 2. Results

### 2.1. Selective Kinase Inhibitors Ipproved for Cncer, Resistance Mechanisms and How Combinations Overcome Resistance

Cancer is often associated with the dysfunction of kinase activity, including receptor and nonreceptor tyrosine kinases as well as serine/threonine kinases. Many drugs that were developed recently are designed to target and inhibit a single kinase (Table 1). One important example is ibrutinib, a small molecule designed to target Bruton’s tyrosine kinase (BTK) that was successfully used in clinical practice for the treatment of chronic lymphocytic leukemia (CLL), mantle cell lymphoma (MCL), and Waldenstrom’s macroglobulinemia. BTK plays a critical role as an effector molecule throughout B-cell development and is crucial in the initiation, survival, proliferation, and progression of B cell lymphoproliferative disorders [7]. In fact, progression-free survival (PFS) rates amongst relapsed/refractory CLL patients treated with ibrutinib were up to 75% at 26 months [8]. While this drug showed high efficacy for many patients, a single point mutation in BTK that causes substitution of serine for cysteine at residue 481 (C481S) results in the inability of ibrutinib to covalently bind to BTK leading to diminished functionality of ibrutinib [8]. In addition to BTK C481S mutations, mutations in PLCg2 were also shown to induce ibrutinib resistance. Three distinct PLCg2 mutations were found in CLL patients with resistance to ibrutinib therapy: arginine-to-tryptophan mutation at position 665 (R665W), leucine-to-phenylalanine at position 845 (L845F), and a serine-to-tyrosine mutation at position 707 (S707Y) [9]. Since PLCg2 is directly downstream of BTK, these gain-of-function mutations in PLCg2 completely bypass the utility of BTK inhibition. Due to these acquired resistance mechanisms, multiple noncovalent BTK-inhibiting drugs such as ARQ-531, vecabrutinib, and pirtobrutinib were developed to specifically target BTK without requiring binding to C481. In addition, multiple combination trials of BTK inhibitors with other targeted therapies were designed to overcome genetic and nongenetic resistance mechanisms.

BRAF is another target for selective kinase inhibitors such as vemurafenib, dabrafenib, and encorafenib. BRAF is part of the MAPK pathway and is responsible for cell division and growth. Driver mutations were described in patients with melanoma [10] as well as patients with nonsmall cell lung cancer (NSCLC) and anaplastic thyroid cancer (ATC). Small inhibitory molecules toward BRAF work by binding to the ATP binding pocket in BRAF and stabilizing it to prevent activation of downstream targets MEK and ERK. They can also increase T-cell infiltration into the tumor by increasing the production of IFNg. These BRAF inhibitors (vemurafenib, dabrafenib, and encorafenib) are specific for the treatment of patients with unresectable BRAF V600E/K/D mutant melanoma [1]. Resistance to these BRAF inhibitors is seen in patients where there is a recovery of the MAPK/ERK or PI3K/AKT signaling especially those that have pre-existing RAS mutations [11]. The activation of these pathways can come from mutations, copy-number alterations, or changes in expression [11]. Currently, the best-known mechanisms to overcome patient resistance to BRAF inhibitors is to combine them with an additional small molecule inhibitor.

A third and equally important group of tyrosine kinase inhibitors are those that target epidermal growth factor receptor (EGFR). EGFR is overexpressed and dysregulated or mutated in many epithelial malignancies. These inhibitors were through several cycles of targeted inhibition followed by developed resistance. For first-generation EGFR tyrosine kinase inhibitors (TKIs), such as gefitinib and erlotinib, their mechanism of action depends on the reversible competitive binding to the ATP binding site of EGFR, and they revolutionized the treatment of NSCLC patients with mutations at L858R and Del19. Unfortunately, within a year most patients develop the resistance mutation EGFR T790M [7]. This was followed by the development of second-generation EGFR/HER TKIs afatinib, dacomitinib, and neratinib engineered to overcome resistance. These inhibitors target EGFR T790M and EGFR WT, however, due to dose-limiting toxicity, many were discontinued in clinical trials [12]. The third-generation EGFR TKIs osimertinib, olmutinib, rociletinib and others were designed to selectively and irreversibly target EGFR T790M as well as activating EGFR mutations, showing promising efficacy in NSCLC resistant to the first- and second-generation EGFR TKIs with lower toxicity observed in patients [12]. From this group of third-generation inhibitors, only osimertinib is currently FDA approved for the treatment of patients with NSCLC. Unfortunately, as seen in many other single-kinase inhibitors, select point mutations are causing resistance in patients currently being treated with osimertinib. Among the mutations seen, C797S in exon 20 of EGFR is currently the most common mechanism of resistance seen in patients. In addition, to select point mutations, acquired EGFR-independent mechanisms of resistance such as NRAS E63K and KRAS activating mutations were observed for multiple third-generation EGFR inhibitors [12]. Future development of EGFR inhibitors is still being explored and developed with possible new fourth-generation inhibitors identified in high-throughput screening assays [13].

**Figure 1:**
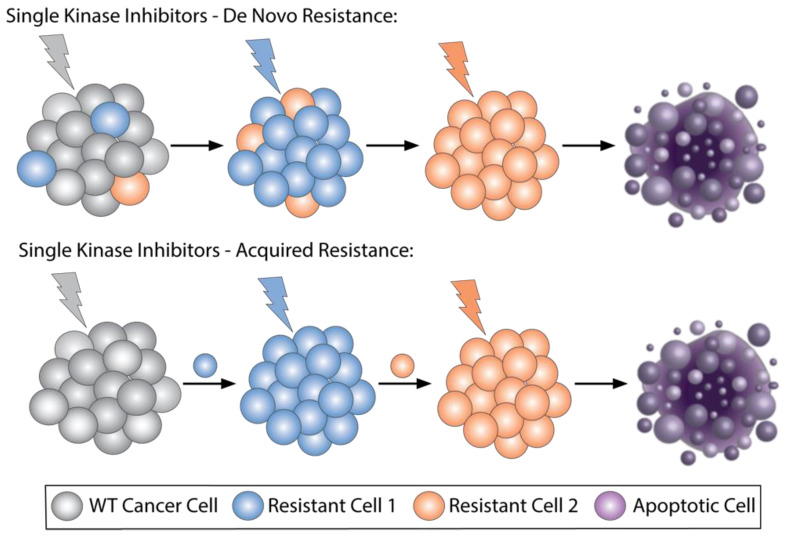
Types of resistance mechanisms occurring in patients. Resistance to targeted therapies can be broadly classified as either de novo or acquired resistance. In case of de novo resistance (top), pre-existing mutations exist in tumor cells that are selected for after treatment (blue colored cells). This could potentially be overcome by treating with sequential selective kinase inhibitors that target escape mechanisms of resistance (orange-colored cells). In acquired resistance (bottom), cells with resistance arise during treatment and cause relapse. Sequential treatments may overcome resistance if given in correct order.

Overcoming resistance mechanisms for the single-kinase inhibitors was a continuously evolving field of research. There are several unique mechanisms that can cause patients to develop resistance to therapy; therefore, this is not a one-size-fits-all solution (Figure 1). Some combinations appear to be quite effective in a majority of patients and could serve as a general treatment strategy. One example of a combination approach that has shown promising results for CLL patients is using the BTK inhibitor ibrutinib in combination with the BCL2 inhibitor venetoclax. Venetoclax is a selective and potent BCL2 inhibitor that induces apoptosis in cancer cells. The combination of these two drugs showed no residual disease after treatment for most patients as well as no unexpected toxicity from combining the two drugs [14]. This combination was given to treatment naïve high-risk and older CLL patients. Each patient in the clinical studies of this combination had one of the following features: chromosome 17p deletion, mutated TP53, chromosome 11q deletion, unmutated IGHV, or an age of 65 years or older [15]. Today, this combination approach is currently used for treatment of select patients while other combination treatment options continue to be explored.

Another exciting example of combination drug therapy to overcome resistance is the combination of the BRAF inhibitor dabrafenib with mitogen-activated protein kinase (MEK) inhibitor trametinib. By approaching BRAF-driven cancers by inhibiting both BRAF and MEK simultaneously, emergence of resistance significantly decreased [16]. This treatment combination was also shown to deliver better responses than monotherapy. Despite these promising results, acquired resistance continues to be a concern for patients. It is possible that inhibition of other host factors in combination with BRAF/MEKi will be required, hinting at the idea of specialized and individualized therapeutics for each patient.

With all the mechanisms of resistance seen in monotherapeutic approaches to inhibit EGFR, it is no surprise that researchers were working diligently to find a combination approach to overcome said resistance. Amongst others, some combination approaches include combining third-generation EGFR inhibitors with MEK/MET inhibitors, cytotoxic chemotherapy, radiation, immune checkpoint inhibitors, as well as monoclonal antibodies [13]. One promising result was observed in the combination of either afatinib or EAIO45 (a fourth-generation EGFR inhibitor) with monoclonal EGFR antibody cetuximab. Cetuximab works by blocking EGFR dimerization and when combined with EAIO45 showed antiproliferative responses both in vivo and in vitro models of lung cancer driven by EGFR L858R/T790M and by EGFR L858R/T790M/C797S mutations [12,13]. EAI045 is currently being studied as both a monotherapy and a combination therapy and is currently considered a novel inhibitor that can overcome both EGFR T790M and C797S mutations [13].

Due to their selectiveness and specificity, single-kinase-inhibiting drugs are often easier on patients (causing fewer side effects); however, the continuous discovery of point mutations that allow for resistance to these agents was discouraging [17]. While these drugs are designed to inhibit single-kinase targets, there are often off-target effects observed for other kinases that might share a similar ATP binding domain. There is also evidence that these inhibitors have nonkinase targets including tubulin and bromodomain and extra-terminal domain (BET) proteins [18]. Overall, it does appear that targeting acquired resistance mechanisms with combinatory therapeutic approaches shows high efficacy and gives us a glimpse into future directions for kinase inhibitors.

**Table 1:** Single kinase inhibitors currently available.

Chemical Structure:	Drug Name:	Target:	Generation:	Known Resistance Mechanism:	Reference:
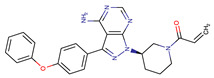	Ibrutinib	BTK	II	BTK mutation (C418S) PLCγ2 mutations (R665W, L845F, (S707Y)	[2,7]
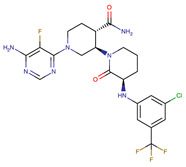	Vecabrutinib	BTK	II	N.A.	[1,2,6]
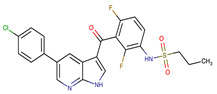	Vemurafenib	BRAF	II	Loss of NF1 or CUL3, VEGFR-1 up-regulation, CRAF up-regulation, BRAF amplification, BRAF N-terminal truncation (splice variant), MCF2 and VAV1 overexpression, EGFR–SFK–STAT3 signaling pathway increased activity, BOP1 loss, Akt signaling activation, ALK activation, COT/TPL2 expression, downregulation of the ubiquitin ligase RNF125, RTK (EGFR and c-MET) upregulation, MAPK, PI3K/AKT and SRC signaling networks activation, CD271 upregulation	[1,6,10]
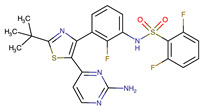	Dabrafenib	BRAF	II	BRAF amplification, BRAF N-terminal truncation, KRAS mutations, N-RAS or MEK1/2 activation, reactivation of MAPK/Erk, alteration to members of the RAS/RAF/MEK/Erk signaling cascade	[1,6,10]
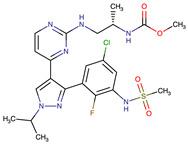	Encorafenib	BRAF	II	BRAF amplification, BRAF N-terminal truncation, alteration to members of the RAS/RAF/MEK/Erk signaling cascade	[1,6,10]
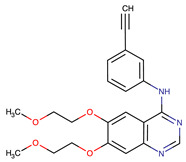	Erlotinib	EGFR	I	EGFR T790M mutation	[12]
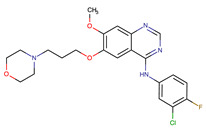	Gefitinib	EGFR	I	EGFR T790M mutation	[12]
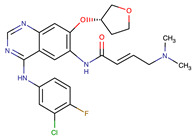	Afatinib	EGFR	II	NRAS amplification or activating mutations KRAS amplification or activating mutations	[12,13]
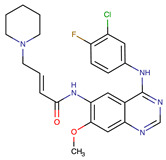	Dacomitinib	EGFR	II	NRAS amplification or activating mutations KRAS amplification or activating mutations	[12]
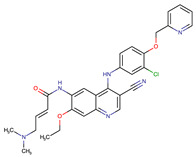	Neratinib	EGFR	II	N.A.	[12]
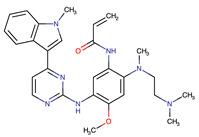	Osimertinib	EGFR	III	EGFR C797S mutation EGFR L798Q mutation NRAS amplification or activating mutations KRAS amplification or activating mutations HER2 *and* MET amplification	[12]
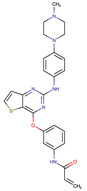	Olmutinib	EGFR	III	EGFR C797S mutation	[12]
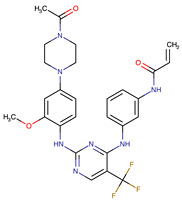	Rociletinib	EGFR	III	EGFR L798I mutation HER2 and MET amplification	[12]
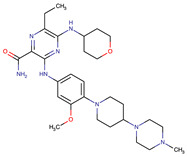	Gilteritinib	FLT3		IRAK1/4 hyperphosphorylation	[19,20]
N.A.	EAIO45	EGFR	IV	N.A.	[12,13]

BTK: Bruton’s Tyrosine Kinase, PLCg2: Phospholipase Cgamma2, RAF: Rapidly Accelerated Fibrosarcoma, BRAF: Rapidly Accelerated Fibrosarcoma homolog B, CRAF: Rapidly, Accelerated Fibrosarcoma homolog C, EGFR: Epidermal growth factor receptor, NRAS: Neuroblastoma RAS viral oncogene homolog, KRAS: Kirsten rat sarcoma virus, NF1: Nuclear factor I, CUL3: Cullin 3, VEGFR-1: Vascular endothelial growth factor receptor 1, MCF2: MCF.2 cell line-derived transforming sequence, VAV1: vav guanine nucleotide exchange factor 1, SFK: Src family of Kinases, BOP1: Block Of Proliferation 1 Protein, ALK: Anaplastic Lymphoma Receptor Tyrosine Kinase, COT/TPL2: Cot (Cancer Osaka Thyroid)/Tumor progression locus 2, MEK: Mitogen-activated protein kinase, ERK: extracellular-signal-regulated kinase, HER2: human epidermal growth factor receptor 2, MET: mesenchymal epithelial transition factor, IRAk1/4: interleukin-1 receptor-associated kinase 1 and 4.

### 2.2. Multikinase Targeted Therapies That Are Approved in Cancer and Potential Drawbacks

**Figure 2:**
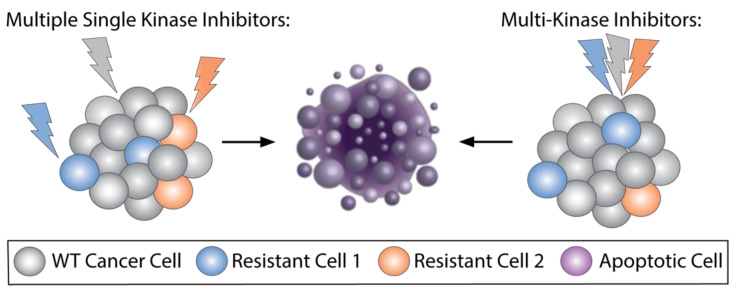
Hitting multiple targets at once to overcome resistance. Since resistance to targeted therapies can occur from residual tumor cells that are not eradicated selective single kinase inhibitors, multiple kinase targeted therapies can be utilized to overcome resistance. Using two or more single kinase selective agents (left) or multikinase inhibitor single agents (right) can target driver mutations and potential escape mechanisms simultaneously and obviate need for multiple sequential therapies.

Cancer is rarely thought to be the product of a single abnormal signaling pathway, making targeting multiple pathways an appealing strategy for tumor eradication. Multitarget drugs also allow for a single drug to be used for multiple indications. Additionally, this strategy can also curb resistance mechanisms that may arise when using selective single-kinase inhibitors (Figure 2). The trade-off, however, may be the increase in adverse effects that comes with inhibiting multiple biologic pathways, many times being important in normal cellular function as well.

One of the earliest and most well-known developments in small molecule protein kinase inhibition was that of imatinib. Imatinib is a tyrosine kinase inhibitor (TKI) that was approved by the FDA in 2001 for the treatment of Philadelphia chromosome-positive (Ph+) chronic myelogenous leukemia (CML) where a characteristic cytogenetic translocation t (9;22) leads to the rearrangement of the breakpoint cluster region (BCR) and Abelson (ABL) genes. This fusion eliminates the inhibitory domain of the ABL protein kinase and causes its constitutive activation and stimulation of multiple signaling pathways. Imatinib’s efficacy in CML relies on the inhibition of BCR-ABL protein kinase; however, it is a nonselective, multikinase inhibitor of BCR-ABL, platelet-derived growth factor receptor (PDGFR), and Kit protein-tyrosine kinases. Imatinib is thus approved for multiple indications such as Kit-mutant gastrointestinal stromal tumors (GIST), systemic mastocytosis, Ph+ acute lymphoblastic leukemia, myeloproliferative neoplasms with PDGFR gene-rearrangement, and hypereosinophilic syndrome with FIP1L1-PDGFRa. The most common mechanisms of resistance to imatinib in CML involve point mutations in four amino acids T315I, T253F, E255K/V, and M351T [21]. T315I disrupts the hydrogen bonding with imatinib; however, the mechanisms of resistance with the other point mutations are less clear. Other mechanisms of resistance involve upregulation of other kinase pathways such as RAS and MEK [22]. While some of the second-generation TKIs can be used in imatinib resistant disease, ponatinib, the third-generation multikinase TKI is the only one effective in T315I mutants due to improved binding of the ponatinib to the ATP binding site [23,24]. In GIST, imatinib resistance can occur in tumors possessing wild-type KIT and/or gain-of-function mutation in PDGFRa. The gatekeeper mutation T670I can disrupt the hydrogen bonding of imatinib, conferring secondary resistance in GIST [22]. Sunitinib, which also targets VEGF in addition to KIT and PDGFR, is active in imatinib resistant GIST, and furthermore, regorafenib is approved for third line therapy. Additional targets of regorafenib are RET, FGFR, and BRAF [25].

Midostaurin, formerly PKC412, inhibits PDGFR, protein kinase C (PKC), spleen associated tyrosine kinase (SYK), proto-oncogene c-Kit, cellular Src kinase (SRC), and vascular endothelial growth factor receptor (VEGFR)-1/-2. Acute myeloid leukemia (AML) patients with fms-like tyrosine kinase 3 (FLT3) internal tandem duplication (ITD) mutations generally have a worse prognosis, including shorter complete remission (CR) duration and higher rates of recurrence when compared to patients without these mutations. Midostaurin is approved for systemic mastocytosis and remains the sole agent approved in combination with induction and consolidation chemotherapy for newly diagnosed FLT3-mutated AML patients [25]. Its inhibitory effects on mutant FLT3 come from the ability to decrease FLT3 autophosphorylation, antagonizing downstream p38 MAPK and STAT5 signaling. Interestingly, studies demonstrated comparable FLT3 inhibitory activity amongst mutant variants, but 10-fold lower inhibition of wild-type FLT3 [26,27]. One of the first point mutation mechanisms discovered to confer midostaurin resistance was N676K, and F691I/L serves as a gatekeeper mutation [28]. Furthermore, the variant FLT3-ITD627E was discovered to cause primary resistance to midostaurin due to upregulation of the antiapoptotic pathway [28]. However, in general, resistance to FLT3 inhibition may be multifactorial with a number of both on- and off-target mechanisms being described [28].

Sorafenib inhibits KIT, VEGFR -1/2/3, FLT3, RET, RAF, and PDGFR [1,6,29]. It was first approved for advanced renal cell carcinoma; however, it is now approved for thyroid and hepatocellular carcinoma (HCC) [30,31,32]. The antiangiogenic properties through inhibition of VEGFR and PDGFR in endothelial cells are thought to be the key to its efficacy, but sorafenib has also been shown to demonstrate apoptotic effects on cancer cell lines through regulation of MCL-1 and the BCL-2 family of proteins [33,34]. Sorafenib resistance was most explored in the realm of HCC. A single most common resistance mechanism has not been well defined, but instead there exist several signaling pathways and characteristics of the tumor microenvironment implicated in the mechanism of sorafenib resistance in HCC [35]. There is ongoing interest in sorafenib as an FLT3 inhibitor for AML; however, it is not approved for this indication.

Other examples of multikinase inhibitors are larotrectinib and entrectinib. Both are receptor tyrosine kinase (RTK) inhibitors of tropomyosin receptor kinase (TRK) however, entrectinib also inhibits ROS proto-oncogene 1 (ROS1). The development of entrectinib was revolutionary in treating ROS1-driven NSCLC. However, patients ultimately relapse within a few years, with the disease being significantly resistant to ROS1-TKIs. The most common mechanism of resistance arises from solvent front mutations (SFM) [36]. Such SFMs include ALKG1202R in ALK-rearranged tumors, ROS1G2032R and ROS1D2033N in ROS1-rearranged tumors and TRKAG595R and TRKCG623R in NTRK1- and NTRK3-rearranged tumors, which were reported after treatment with entrectinib (TRKA/B/C, ROS1, and ALK inhibitor) and larotrectinib (TRKA/B/C inhibitor) [36,37]. These two kinase inhibitors have off-target resistance mechanisms including genetic alterations in other RTKs or downstream pathway members [38]. Interestingly, these fusion mutation mechanisms of resistance, both on target and off-target, were described analogously in other cancer types such as ALK fusion-positive lung cancer and ROS1 fusion-positive lung cancer [39]. Due to their smaller size, selitrectinib and repotrectinib are currently being tested as second-generation TRK inhibitors that can engage with the ATP binding pocket while overcoming on-target resistance caused by steric hindrance [37,40].

One trade off to multikinase inhibition however, may be the increase in adverse effects and drug reactions. The earlier-mentioned drugs (Table 2) all have a wide spectrum of side effects that can sometimes hinder use or compliance in the clinical setting. Given their mechanisms of action, many side effect profiles overlap and include nausea, vomiting, diarrhea, skin rash, hand-foot rash syndrome, myalgias, joint pain, fatigue, and headaches, for example. Some of the second and third generations of the BCR-ABL TKI family of drugs beyond imatinib have additional specific side effect profiles including fluid retention including effusions, and cardiovascular risks. While most side effects can be managed with supportive measures, the diverse side effect profile of multitargeted kinase inhibition might make combinations with other targeted therapies more challenging.

### 2.3. Ongoing Trials of Combining Two Selective Targeted Agents, Dual-Targeted Agents, or Multikinase Inhibitors

The increase in knowledge in various signaling pathways is evident in the array of novel targets under development over the last decade, in both hematological malignancies and solid tumors. These targets are involved in important pathways that are crucial for tumor survival and proliferation, including prosurvival, antiapoptotic, and epigenetic modification pathways [48]. Combination therapy, simultaneously utilizing two or more target inhibitors, may increase treatment efficacy, depending on the molecular landscape, through additive or synergistic effects (Table 3).

CG-806 is an oral, noncovalent, and potent inhibitor for both wild-type and several mutant forms of BTK, including the C481S mutation, which renders first-generation covalent BTK inhibitors ineffective [28], as well as all known forms of FLT3 receptor tyrosine kinase mutations. As mentioned previously, BTK is an effector molecule in B-cell development while FLT3 is a class III receptor tyrosine kinase that is important for hematopoietic stem cell growth and differentiation [49]. Activation of any of the three known gene mutations: FLT3-ITD point mutations in the activation loop of the second tyrosine kinase domain (FLT3-TKD) and point mutations in the juxtamembrane can prompt factor-independent cellular growth as well as activate various downstream signaling pathways. This leads to the propagation of cellular dysregulation and transformation of hematopoietic stem cells to myeloproliferative disease [49]. Due to its versatility, CG-806 is currently undergoing Phase 1a/b trial (NCT04477291) in patients with relapsed/refractory AML as well as Phase 1a/b trial (NCT03893682) to evaluate its safety and tolerability in patients with CLL/SLL or Non-Hodgkin’s Lymphomas. Preliminary results in the CLL/SLL and Non-Hodgkin’s lymphoma (NHL) patients who previously failed ibrutinib, rituximab, venetoclax, and other therapies, show no dose-limiting toxicities and consistent tolerability [50]. The ability of CG-806 to theoretically suppress multiple oncogenic signaling pathways will ideally be efficacious in B-cell malignancies refractory to single target inhibitors [50]. Further studies utilizing CG-806 in combination with venetoclax show inhibition of driver and rescue pathways within in-vivo studies using double/triple hit lymphoma cell lines that harbor MYC/BCL2/BCL6 mutations, yielding promising results [51].

Repotrectinib (TPX-0005) is a next-generation receptor tyrosine kinase (ROS1), pan-TRK, and anaplastic lymphoma kinase (ALK) TKI inhibitor, created to overcome refractory SFMs which often occur in patients with ROS1/NTRK/ALK-rearranged malignancies, such as NSCLC. Repotrectinib is currently undergoing Phase1/2 trial (NCT03093116), TRIDENT-1 trial, in its administration to patients with advanced solid tumors harboring ALK, ROS1, or NTRK1-3 rearrangements, and observation of its antitumor activity, safety, and tolerability. It was specifically created to overcome refractory SFMs that relapse on available TKIs. So far, the trial has demonstrated a response rate in 50% of patients within NTRK-positive, TKI-treated tumors [41,42], prompting the FDA to grant fast track designation to repotrectinib for treatment of patients with advanced solid tumors with NTRK gene fusion who progressed on at least 1 prior line of TRK TKIs.

Dual targeting of FLT-3 and IRAK with dual kinase inhibitors has begun to spark interest in its ability to block innate immune signaling, resulting in the prevention of adaptive resistance of FLT3-ITD AML to FLT3 inhibitors. Though there are two FDA-approved agents for the inhibition of FLT3, midostaurin for newly diagnosed AML and gilteritinib for relapsed/refractory AML, there continues to be an overall poor prognosis associated with FLT3-ITD malignancies. Interestingly, it was discovered that mediation of primary adaptive resistance to FLT3 inhibitor was through a non-FLT3-mediated cell-intrinsic mechanism rather than directly acquired FLT3 mutations. There is an increase in phosphorylated interleukin-1 receptor-associated kinase (IRAK)1/4 in response to FLT3 inhibition (gilteritinib or quizartinib), leading to compensatory activation of innate immune stress pathways that elicit adaptive resistance to FLT3 inhibitors. IRAK is a serine/threonine kinase associated with the IL-1 receptor, which harbors the Toll/IL-1 receptor (TIR) domain. Upon MyD88-dependent activation of toll-like receptors (TLR), IRAK-1, IRAK-1, and TRAF6 are recruited to the receptor, prompting phosphorylation of IRAK-1 by IRAK-4 [19,20,52], eliciting innate immune responses through inflammatory cytokine production. Recently, it was shown that TLRs play a crucial role in complex interactions at the innate and adaptive immune interface, modulating adaptive immune responses through dendritic cell maturation and Th1/Th2 polarization amongst CD4+ helper T cell responses [53]. Mechanisms of excessive IRAK1/4 activation may include increasing expression of TLR 9 with subsequent amplification of innate immune pathways in FLT3-ITD AML cells and/or arising genetic mutations within the spliceosome (SF3B1 or U2AF1) which causes constitutive activation of the “myddosome”, leading to NF-kB overactivity and excessive B-cell proliferation, which can cause IRAK-4-L overexpression.

Studies were performed inhibiting both FLT3 and IRAK1/4, which demonstrated a synergistic effect on cell growth inhibition in vitro studies with NCGC1481 [49,54]. NCGC1481 is a dual FLT3/IRAK inhibitor proven to be more effective at preventing IRAK1/4 activation when compared to two separate inhibitors of FLT3 and IRAK1/4 given simultaneously [55]. CA-4948 is a small molecule inhibitor of interleukin-1 receptor-associated kinase 4 (IRAK4) and FLT3 and is currently undergoing a Phase 1/2 study (NCT03328078) for its efficacy either alone as a monotherapy or in combination with ibrutinib in patients with relapsed or refractory hematologic malignancies, including NHL, CLL, and Waldenstrom’s macroglobulinemia (WM). Interestingly, the study will evaluate CA-4948 as both a monotherapy and in combination with ibrutinib. CA-4948 is also being tested in AML (NCT04278768) and so far, preliminary data show 4 of 4 patients with FLT3 and spliceosome mutations treated with monotherapy had an objective response with a decrease in marrow blast percentage. Nine out of 11 patients without spliceosome/FLT3 mutations achieved tumor reduction when CA-4948 was added to venetoclax or azacitidine [56].

Aside from multikinase inhibitors or dual-targeted agents, combining single-targeted therapeutics has also been a strong area of interest. In particular, ruxolitinib (INCB018424), a potent, selective oral inhibitor of JAK1/JAK2 was a drug of interest to be paired with another selective inhibitor, some of which include IFNγ2 (PEG-IFNγ2), antifibrotic agents (anti-LOXL2), Bcl-2/Bcl-xL inhibitors, DNA methyltransferase inhibitors (azacitidine), and Hedgehog Pathway Inhibitors [46]. The JAK-STAT (Janus kinase-signal transducer and activator of transcription) pathway is theoretically simple, consisting of activation of type I and II cytokine receptors, which leads to JAK transphosphorylation and subsequent recruitment of various STATs to be phosphorylated. These STATs then dimerize and travel to the nucleus, where it regulates transcription of a multitude of genes, playing a major role for a wide range of crucial biological processes like cellular proliferation, differentiation, and immune regulation [57]. JAK/STAT activating mutations are found in 50–95% of patients with myeloproliferative neoplasms, polycythemia vera (PV), essential thrombocytosis (ET), and primary myelofibrosis (PMF), particularly in JAK2 (valine to phenylalanine change, V617F), and now play an increasing evident role in other hematologic malignancies as well. In particular, high STAT3 expression is able to reduce dampen the expression of genes involved in glycolysis, allowing for tumor cells to grow in a hypoxic environment [47]. Ruxolitinib, now FDA approved for PV, MPN, and acute graft-versus-host disease, is being paired with thalidomide as a combination targeted therapy, currently undergoing phase II trial for patients with primary, postpolycythemia vera, or postessential thrombocythemia myelofibrosis (PMF, post-PV MF, or post-ET MF) (NCT03069326).

Thalidomide targets cereblon (CRBN), a ligand-dependent substrate receptor of the E3 ubiquitin ligase complex cullin-RING ligase 4 (CRL4CRBN) [58]. It was shown in studies to elicit a response in 30–50% of patients with multiple myeloma as a single agent and to decrease transfusions in patients with myelodysplastic syndrome (MDS) [58]. More recently, thalidomide derivatives, immunomodulatory imide drugs (IMiDs), were shown to have potent antitumor activity. When thalidomide binds to CRBN, CRBN can target multiple neosubstrates, such as casein kinase 1 alpha (CK1α, a CK1 protein that regulates cell cycle signaling/apoptosis) or Ikaros (lymphoid transcription factor essential of myeloma cell survival), which allows for inhibition of angiogenesis and suppression of cellular proliferation [58]. This trial is currently ongoing as a multicenter, two stage phase II trial designed to assess the efficacy of this drug in PMF, post-PV MF, or post-ET MF through 6 cycles of oral therapy.

Another more recent investigational drugs in the multikinase inhibitor domain are TGO2 or zotiraciclib. TGO2 is an inhibitor of both transcriptional and cell-cycle regulator cyclin dependent kinases (CDK), in addition to JAK2 and FLT3. TGO2 gained interest as a therapeutic agent in AML, CLL, and gliomas based upon preclinical and phase I trials [35,43,44,59]. When compared to inhibitors that block only one kinase target, CKD, JAK2, or FLT3, TGO2 exhibited increased potency with significantly lower IC50 in cell assays composed of various solid and liquid tumor lines. The same was seen in populations of AML blasts when compared to other FLT3 inhibitors [43,60]. Given the multifaceted inhibitory profile of TGO2, the mechanisms underlying its clinical efficacy are similarly diverse. Studies suggest TGO2 inhibits cell proliferation and induces cell death by blocking RNA transcription and downregulating antiapoptotic proteins. TGO2 is being studied in combination with temozolomide in recurrent high-grade gliomas and is currently in phase 1/2 trial (NCT02942264) [44,60].

Whether it is a single multikinase inhibitor or combination of single-target therapies, inhibiting multiple signaling pathways were shown in many instances to have additive or synergistic effects. This not only reduces possibility of drug resistance, it also can halt mitotic division stunting cancer stem cell proliferation, slow tumor growth and metastatic potential, and downregulate autocrine growth factors.

### 2.4. Immunotherapies, Cancer Stem Cells, Degraders, and Drugs That Target Multiple Pathways

In addition to the above strategies focused on kinase inhibition, other forms of targeted therapy were recently approved and are advancing alongside them. These approaches involve harnessing the immune system against cancers, targeting cancer stem cell regulators, degrading proteins rather than inhibiting them, and targeting proteins that affect multiple pathways such as epigenetic regulators. Immune checkpoint inhibitors were shown to be very effective in cancer treatments. PD-1/PD-L1 and CTLA-4 inhibitors were the first to gain approval. Pembrolizumab, a PD-1 inhibitor was the first drug ever to receive a tumor-agnostic approval for patients with mismatch repair deficient (MMRd) solid tumors. Rather than development of resistance, the challenge for immunotherapies is predicting the population of patients who will respond [61]. Even the above-mentioned biomarker of MMRd tumors is not universal. Therefore, combinations of two immune checkpoint inhibitors or immunotherapy plus other targeted agents is being explored. Other immunotherapy targets such as TIM-3 and LAG-3 inhibitors are being developed [62]. Additionally, the interplay between cancer and the immune system as well as the surrounding tumor microenvironment is an area still ripe for further discoveries [62].

Cancer stem cells (CSCs) contribute to the initiation, reoccurrence, and metastasis of cancer [4,63]. Due to their plasticity and tumorigenic properties, CSCs often lead to relapse [64,65]. Additionally, CSCs have also been associated with chemotherapy resistance in many patients [63,64]. Most treatments offered target proliferating cells but have little or no effect on CSCs, causing tumor shrinkage without complete eradication of the cancer [63,64]. With this in mind, the field is moving towards developing more specific therapies to target CSCs [63,65]. In conjunction with currently offered cancer therapies, CSC targeting drugs could lead to better outcomes and fewer relapses in patients [63]. While there are no currently approved drugs that specifically target CSCs directly, the development of small molecules that target crucial CSC pathways such as Wnt, Hedgehog, Notch, Hippo, and autophagy are currently being pursued in several cancer types [63,64,65,66].

In addition to the above-mentioned pathways, researchers are also investigating the tumor microenvironment (TME) of cancer stem cells. Recently, there was emerging evidence showing that the TME is involved in regulating tumor plasticity [66]. The TME can activate multiple signaling pathways that not only support CSCs “stemness” but also promote immune escape [66,67]. Within the TME there are various cell types such as immune cells, mesenchymal stem cells (MSCs), and cancer associated fibroblasts (CAFs) that together provide an immunosuppressive environment and support CSC survival [67,68]. A better understanding of CSCs and how to target them in combination with currently offered treatments could become pivotal in overcoming current drug resistance in cancer in years to come [63,64,65,66].

In the last few years, a novel approach to target proteins in cancer was developed not by blocking their enzymatic activity but in reducing their levels by targeted degradation [69]. This goal is similar to those of siRNA or genome engineering [70], but there are still tremendous limitations to those approaches, [71,72,73] such as nonreversibility of gene editing. This new strategy of targeted protein degradation uses small molecules (taking advantage of their oral availability) but tag them with unique cellular signals able to trigger an intracellular degradation process of the bound protein via the ubiquitin-proteasomal machinery [74]. The main advantage of this approach is that now the small molecules available to target a specific protein wouldn’t be limited only by their ability to block its biological activity but could also target domains involved in trafficking and scaffolding, broadening the spectrum of compounds accessible to be used as drugs, since the minimum requirement would be for the ligand to bind specifically [75].

One of these methods is PROTAC - proteolysis targeting chimeras [74]. As of today, there are multiple clinical trials involving targeted degraders [76]. For example, ARV-110 targets the androgen receptor and is now in phase II clinical trial for treating metastatic castration resistant prostate cancer (NCT03888612) [76]. CFT7455 targets Ikaros and Aiolos, two zinc-finger transcription factors that are regulators of lymphoid development and differentiation and is in phase I trials for the treatment of refractory NHL or multiple myeloma (NCT04756726) [77]. While this approach is exciting and may allow drugging the “undruggable”, the problem of selecting cancer cells with mutations in the binding area of the compound will still exist [78].

Epigenetic drugs have the ability to affect multiple pathways at once by affecting gene regulation. Some examples include DNA methyltransferase inhibitors and histone deacetylation inhibitors. Cancers also have recurrent mutations in epigenetic regulators themselves and different epigenomes than their healthy counterparts [79]. With some exceptions the epigenetic drugs have not had widespread utility across cancers, but the field is relatively new and very recent compounds such as PRMT5 inhibitors are showing great promise in clinical trials [80].

Other agents such as nuclear export inhibitors target cellular processes that have broad effects but appear to affect cancer cells preferentially [81]. XPO1 has a major role in cellular homeostasis by transporting proteins and RNAs from the nucleus to the cytoplasm and is the sole exporter of well over 200 proteins [82]. Selinexor is an inhibitor of XPO1 and was approved by FDA for use as a treatment for refractory multiple myeloma and lymphoma. The role of selinexor and second generation XPO1 inhibitors in other cancer types and in combinations is still being examined [83,84]. Thus far, no resistance mechanisms to selinexor were described. It could be speculated that targeting multiple pathways by selecting a target with pleiotropic effects, cancers may be unable to mount resistance without triggering programed cell death.

## 3. Discussion

Each of the kinase inhibitor groups discussed here are meant to serve as a brief overview as they could easily each become their own independent review and this review focused on the pros and cons of different targeted therapeutic strategies rather than exhaustively covering all targeted therapeutics (Table 4). In reality, the complexity of cancer will likely require mixing and matching these approaches discussed above in different disease types and settings. However, the complexity within individual cancers (intra-tumor heterogeneity) was already proven to provide resistance to most attempts to target individual protein kinases [85,86,87]. This suggests at least some combination strategy will need to be employed to successfully eradicate residual tumor cells that can serve as reservoirs for resistance and relapse. Specificity sounds attractive for drug development; however, most cancers have multiple aberrant pathway activation and can often find simple ways to evade very selective targeted inhibition [88,89]. Newly developed techniques using multiplex gene expression analysis were designed to screen chemical libraries for cancer specific proteins to help develop several new classes of multikinase inhibitors [90]. Additionally, new in-silico models using multiomics data are currently being used to help predict synergistic drug combinations [91]. With the rapid development of these technologies future cancer treatment regimens could be rationally designed to more effectively eradicate tumor cells and offer more cures to patients.

## Figures and Tables

**Table 2:** Multikinase inhibitors currently available.

Chemical Structure:	Drug Name:	Target:	Generation:	Known Resistance Mechanism:	Reference:
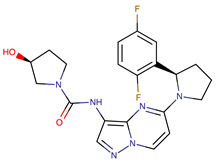	Larotrectinib	TRKA, B and C	I	G623R, G696A, and F617L mutations in the kinase domain	[36,37,38]
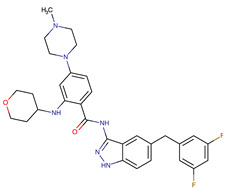	Entrectinib	TRKA, B and C ROS1 ALK	I	G623R, G696A, and F617L mutations in the kinase domain L1951R, S1986Y/F, F2004V, L2026M, and G2032R mutations in the kinase domain Activation of the EGFR, RAS or KIT signaling pathways C1156Y, L1196M, and G1269A (C1156Y/G1269A) mutations altering the ATP-binding pocket Activation of the EGFR, cMET, KRAS, or AXL signaling pathways	[36,37,38]
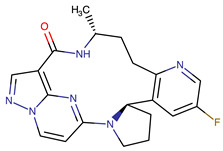	Selitrectinib	TRKA, B and C	II	Kinase domain mutation G667C	[37,40]
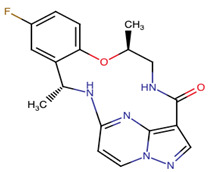	Repotrectinib	ROS1, TRKA, B and C, ALK	II	N.A.	[37,40,41,42]
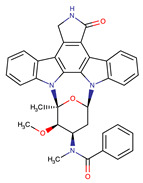	Midostaurin	PDGFR/PKC/SYK/c-Kit/SRC/VEGFR/FLT3	I	N676K and F691I/L mutations in FLT3	[19,20,25,26,27,28]
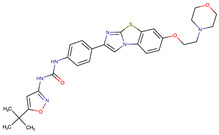	Quizartinib	FLT3	II	FLT3 F691L, D835F/V/Y and Y842C/H mutation KIT D816 mutation	[19,20]
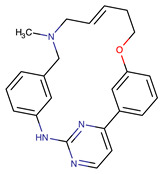	Zotiraciclib	CDK9/JAK2/FLT3	I	Compensatory induction of MYC expression	[43,44,45]
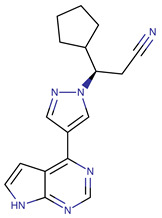	Ruxolitinib	JAK1/JAK2	II	Reactivation of JAK/STAT signaling via heterodimer formation between JAK2 and JAK1 or TYK2 Paracrine protective effects by cytokines (interleukin-6, fibroblast growth factor) Activation of alternative kinases not inhibited by ruxolitinib (MEK/ERK) Epigenetic mutations Mutations in JAK2 Y931C, G935R, R938L, I960V and E985K (in vitro only)	[46,47]
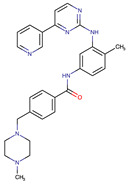	Imatinib	ABL1/PDGFR/cKIT	I	Mutations within the Abl kinase domain/Overexpression of Bcr-Abl/Src activation	[21,22,23]
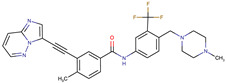	Ponatinib	ABL1,FGFR, PDGFR, SRC, RET, KIT, and FLT1	III	BCR/ABL compound mutations: T315I/F359V, E255V/T315I, T315I/F359C, T315I/E453K	[21,22]
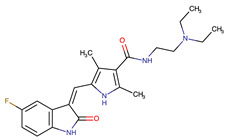	Sunitinib	VEGF/KIT/PDGFR	I	Androgen receptor (AR) expression EIF3D or EZH2 overexpression EGFR activation	[25]
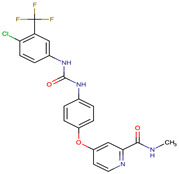	Sorafenib	c-KIT, VEGFR -1/2/3, FLT3, RET, RAF, and PDGFR	I	Overexpression of both αB-Crystallin and 14-3-3ζ GRP78 overexpression	[29,30,31,32,33,34,35]

TRKA: tropomyosin receptor kinase A, ROS1: ROS Proto-Oncogene 1, ALK: anaplastic lymphoma kinase (Ki-1), AXL: AXL receptor tyrosine kinase, PDGFR: platelet-derived growth factor receptor, IRAk1/4: interleukin-1 receptor-associated kinase 1 and 4, FLT3: Fms-like tyrosine kinase 3, c-KIT: tyrosine-protein kinase KIT, RET: rearranged during transfection/ Proto-Oncogene Tyrosine-Protein Kinase Receptor Ret, GRP78: glucose-regulated protein 78, ABL1: Abelson murine leukemia virus, SRC: proto-oncogene tyrosine-protein kinase Src, FLT1: Fms Related Receptor Tyrosine Kinase 1), FGFR: fibroblast growth receptor (1 to 4), EZH2: Enhancer of zeste homolog 2, EIF3D: (Eukaryotic Translation Initiation Factor 3 Subunit D, JAK1/JAK2: Janus Kinase 1 and 2, CDK9: cyclin-dependent Kinase 9, PKC: protein kinase C, SYK: spleen tyrosine kinase.

**Table 3:** Current combination drug trials using multiple selective inhibitors, dual-targeted therapies, or multikinase inhibitors.

Combination Drugs/Ongoing Trials:	Targets:	Trial Phase	ClinicalTrials.gov Identifier:	Cancer Treated
Ibrutinib + Venetoclax	EGFR + BCL2	2	NCT03045328	Refractory chronic lymphocytic leukemia (CLL) and small lymphocytic leukemia (SLL)
Dabrafenib + Trametinib	EGFR + MEK	1/2	NCT01767454 NCT02296996	Melanoma
Zotiraciclib	CDK, JAK2, FLT3	1/2	NCT02942264	Gliomas
Ruxolitonib + Thalidomide	JAK1/2, CRBN	2	NCT03069326	PMF, post-PV MF, or post-ET MF
CA-4948	FLT3, IRAK	1/2	NCT04278768	AML
CA-4948 + Ibrutinib	FLT3, IRAK BTK	1/2	NCT03328078	NHL, CLL, and Waldenstrom’s macroglobulinemia (WM)
CG-806	BTK, FLT3	1a/b	NCT04477291 NCT03893682	AML, CLL/SLL, NHL
Repotrectinib	ROS1, TRK, ALK	1/2	NCT03093116	NTRK fusion tumors

EGFR: epidermal growth factor receptor, BCL2: B-Cell receptor 2, MEK: mitogen-activated protein kinase, CDK: cycline-dependent Kinase, JAK1/JAK2: Janus Kinase 1 and 2, FLT3: Fms-like tyrosine kinase 3, IRAK: interleukin-1 receptor-associated kinase, BTK: Bruton’s Tyrosine Kinase, ROS1: ROS Proto-Oncogene 1, TRKA: tropomyosin receptor kinase A, ALK: Anaplastic Lymphoma Receptor Tyrosine Kinase.

**Table 4:** Advantages and disadvantages of selective vs. nonselective inhibitors.

	Selective Inhibition	Non-Selective Inhibition
Advantages	▪ Favorable toxicity profiles ▪ Candidates for combination drug investigation ▪ Easy determination of mechanism of action	▪ Approval for multiple indications ▪ Targeting of multiple dysregulated pathways ▪ Less prone to resistance mechanisms
Disadvantages	▪ Narrow scope of approved indications ▪ Decreased ability to overcome resistance mechanisms	▪ Wider toxicity profile ▪ Less easily combined with other drugs due to unpredictable toxicity

## Data Availability

Not applicable.
